# Economic benefit of subcutaneous rapid push versus intravenous immunoglobulin infusion therapy in adult patients with primary immune deficiency

**DOI:** 10.1186/1710-1492-8-S1-A20

**Published:** 2012-11-02

**Authors:** Adriana Martin, Louis Lavoie, Mireille Goetghebeur, Robert Schellenberg

**Affiliations:** 1 SCIG Home Infusion Program, St. Paul’s Hospital, Vancouver, British Columbia, Canada; 2 BioMedCom Consultants inc. , Montreal, Quebec, Canada; 3 Dept. of Medicine, Cardiovascular and Pulmonary Research, University of British Columbia, Vancouver, Canada

## Background

Primary immune deficiencies (PID) are genetic disorders resulting in recurrent infections. Immunoglobulin replacement therapy in PID patients can be achieved intravenously (IVIG) or subcutaneously (SCIG) with similar efficacy and safety profiles but with different resource use and associated costs.

## Methods

SCIG and IVIG options for immunoglobulin replacement therapy in adult PID patients were compared in a cost-minimization model over three years of treatment. The model focused on direct medical costs for infusion supplies and personnel. A three-year budget impact model assessed the economic impact on the healthcare system of switching from IVIG to SCIG for PID patients of the BC Central Transfusion Registry. Sensitivity analyses were performed for both models to measure the effect of different modalities of IVIG treatment and of the proportion of patients switching from IVIG to SCIG.

## Results

The cost-minimization model estimated SCIG treatment cost per patient over three years at $1978 compared to $7714 for IVIG, resulting in savings to the healthcare system of $5736, principally due to reduced hospital personnel costs. This figure varied from $5035 to $8739 for different modalities of IVIG therapy. Assuming that 50% of patients who received IVIG switched to SCIG, the budget impact model estimated cost savings for the first three years at $1,307,894 or 37% of the personnel and supply budget.

## Conclusion

This study demonstrated that rapid push home-based SCIG was less costly than hospital-based IVIG for immunoglobulin replacement therapy. This approach provides not only a beneficial option from the patient perspective but also results in significant savings to the healthcare system for immunoglobulin replacement therapy in adult PID patients in a Canadian context.

